# Representares sociais sobre cateteris mo periférico pediátrico na perspectiva da família e enfermagem

**DOI:** 10.15649/cuidarte.2303

**Published:** 2023-03-29

**Authors:** Paula Krempser, Célia Pereira-Caldas, Cristina Arreguy-Sena, Laércio Deleon-de-Melo

**Affiliations:** 1 Universidade Federal de Juiz de Fora (UFJF), Faculdade de Enfermagem da UFJF FACENF-UFJF, Programa de Pós-Gradua?ao em Enfermagem. Juiz de Fora, MG - Brasil. E-mail: paula@ufjf.br Universidade Federal de Juiz de Fora Universidade Federal de Juiz de Fora Brazil paula@ufjf.br; 2 . Universidade do Estado do Rio de Janeiro (UERJ), Faculdade De Enfermagem da UERJ, Programa de Pós-Gradua? ao em Enfermagem. Rio de janeiro, RJ - Brasil. E- mail: celpcaldas@gmail.com Universidade do Estado do Rio de Janeiro Universidade do Estado do Rio de Janeiro Brazil celpcaldas@gmail.com; 3 Universidade Federal de Juiz de Fora (UFJF), Faculdade de Enfermagem da UFJF (FACENF-UFJF), Aposentada. Juiz de Fora, MG - Brasil. E-mail: cristina.arreguy@ufjf.br. Universidade Federal de Juiz de Fora Universidade Federal de Juiz de Fora Brazil cristina.arreguy@ufjf.br; 4 Universidade Federal de Juiz de Fora (UFJF), Faculdade de Enfermagem da UFJF (FACENF-UFJF). Programa de Pós-Gradua?ao em Enfermagem. Juiz de Fora, MG - Brasil. E-mail: laerciodl28@hotmail.com Universidade Federal de Juiz de Fora Universidade Federal de Juiz de Fora Brazil laerciodl28@hotmail.com

**Keywords:** Nursing, Catheterization, Peripheral, Child Health, Family, Psychology, Social., Enfermería, Cateterismo Periférico, Salud del Niño, Familia, Psicología Social., Enfermagem, Cateterismo Periférico, Saúde da Crianza, Família, Psicologia Social.

## Abstract

**Introdujo:**

a punáo venosa constitui-se num procedimento de atribuido da equipe de enfermagem e destaca-se entre as tecnologias imprescindíveis para garantir a sobrevivencia e a terapia das comorbidades agudas/crónicas infantis. Objetivou-se discutir as representares sociais dos profissionais de enfermagem e acompanhantes sobre a punáo venosa periférica realizada em crianzas hospitalizadas.

**Materiais e métodos:**

pesquisa delineada nas abordagens processual e estrutural da Teoria das Representares Sociais realizada na pediatria de um hospital geral de Minas Gerais em abril/setembro de 2018. Foram coletados dados de caracterizaáo sociodemográficos que foram tratados por estatística descritiva; evocaáo livre de palavras náo hierarquizada analisadas prototípicas e lexicograficamente e; entrevistas individuais em profundidade que foram submetidas a análise de conteúdo. Atendidos todos os aspectos ético-legais.

**Resultados:**

as representares sobre a punáo segundo os profissionais de enfermagem estruturaram-se sobre as dificuldades em realizar o procedimento e na inseráo da máe no cuidado compartilhado e para os acompanhantes foram impactantes negativamente sobre seu psicológico.

**Discussoes:**

as representares sociais da punáo venosa dos profissionais de enfermagem reduziram- se as técnicas de inseráo do cateter, negligenciando o cuidado centrado na família que por sua vez representaram os sentimentos ao verem a crianza puncionada.

**Conclusoes:**

as aproximaóes/distinóes representacionais identificadas entre os subgrupos sobre a punáo nas crianzas contribuíram com reflexóes apontando a necessidade de um redimensionamento sociocultural e humanizado dos cuidados de enfermagem.

## Abstract (en)

**Introduction:**

venipuncture is an attribution procedure of the nursing team and stands out among the essential technologies to ensure the survival and therapy of acute/chronic child hood comorbidities. Objectified to discuss the social representations of nursing professionals and caregivers about peripheral venipuncture performed in hospitalized children.

**Materials and methods:**

research outlined in the procedural and structural approaches of the Theory of Social Representations carried out in pediatrics at a general hospital in Minas Gerais in April/ September 2018. Sociodemographic data were collected and treated by descriptive statistics; free evocation of non-hierarchical words analyzed prototypically and lexicographically and; in depth individual interviews that underwent content analysis. All ethical and legal aspects are met. All ethical and legal aspects are met.

**Results:**

the representations about the puncture, according to the nursing professionals, were structured on the difficulties in performing the procedure and the insertion of the mother in shared care and for the companions had a neg ative impact on their psychological condition.

**Discussions:**

the nursing professionals' social representations of venipuncture were reduced to catheter insertion techniques, neglecting family-centered care, which in turn represented the feelings of seeing the punctured child.

**Con clusions:**

the representational approximations/distinctions identified between the subgroups about puncture in children contributed with reflections pointing to the need for a sociocultural and humanized resizing of nursing care.

## Resumen

**Introducción:**

la venopunción es un procedimiento de atribución del equipo de enfermería y se destaca entre las tecnologías imprescindibles para asegurar la supervivencia y el tratamiento de las comorbilidades infantiles agudas/crónicas. Objetivado discutir las representaciones sociales de los profesionales de enfermería y cuidadores sobre la punción venosa periférica realizada en niños hospitalizados.

**Materiales y métodos:**

investigación esbozada en los enfoques procedi- mentales y estructurales de la Teoría de las Representaciones Sociales realizada en pediatría en un hospital general de Minas Gerais en abril/septiembre de 2018. Los datos sociodemográficos fueron recolectados y tratados mediante estadística descriptiva; evocación libre de palabras no jerárquicas analizadas prototípicamente y lexicográficamente y; entrevistas individuales en profundidad que se sometieron a análisis de contenido. Se cumplen todos los aspectos éticos y legales.

**Resultados:**

las representaciones sobre la punción, según los profesionales de enfer mería, se estructuraron sobre las dificultades para realizar el procedimiento y la inserción de la madre en el cuidado compartido y para los acompañantes incidió negativamente en su condi ción psicológica.

**Discusiones:**

las representaciones sociales de la venopunción de los profesio nales de enfermería se redujeron a técnicas de inserción de catéteres, descuidando el cuidado centrado en la familia, que a su vez representó los sentimientos de ver al niño pinchado.

**Con clusiones:**

las aproximaciones representacionales/distinciones identificadas entre los subgru pos sobre la punción en niños contribuyeron con reflexiones que apuntan a la necesidad de un redimensionamiento sociocultural y humanizado de los cuidados de enfermería.

## Introdujo

A Pungáo Venosa Periférica (PVP) constitui-se num procedimento de atribuigáo da equipe de enfermagem e destaca-se entre as tecnologías imprescindíveis para garantir a sobrevivencia e a terapia das comorbidades agudas/cronicas infantis atrelada as habilidades e competencias específicas quando executada no ambiente hospitalar no público infantil que deve ser acompanhada por um responsável durante o período de internado conforme assegurado em lei^1^^-^^3^.

Conciliar a preferencia do local da pungáo, reunir esforgos e co-participagáo da equipe de saúde, da crianga e do acompanhante, priorizar o conforto e a liberdade da crianga e assegurar uma execugáo técnica exímia, interativa e comunicacionalmente efetiva constitui estratégia tecnológica capaz de minimizar desconfortos^4^, atender as recomendagóes da Agenda Nacional de Prioridades de Pesquisa em Saúde com foco na crianga (eixo 14- saúde materno- infantil^5)^, garantindo a continuidade das atividades diárias e de recreagáo da crianga durante a hospitalizagáo, fato que distingue essa técnica quando executada em adultos/idosos, em virtude da fase de desenvolvimento em que elas se encontram e de vulnerabilidades peculiares a idade^1^^-^^2^.

A seguranga da terapia infusional, justifica-se por reduzir eventos adversos e múltiplas pungóes que impactam sobre o significado e as vivencias infantis e dos acompanhantes sobre uma internagáo hospitalar e ainda no desenvolvimento e crescimento da crianga após a alta hospitalar^2^^,^^6^ no contexto das internagóes. Tal fato justifica a necessidade em se discutir as Representagóes Sociais dos profissionais de enfermagem e acompanhantes sobre a PVP realizada em criangas hospitalizadas visando a captagáo das construgóes simbólicas consideradas do “senso comum” na perspectiva da Teoria das Representagóes Sociais (TRS^7),(^^8^, mediante a existencia de uma lacuna literária em relagáo a temática.

Dessa forma, as Representagóes Sociais (RS) da PVP em criangas elaboradas pelos profissionais de enfermagem e acompanhantes constituiu-se no objeto de investigagáo que se propóe a contribuir para que seja preenchida a lacuna identificada na literatura no que tange a superagáo da fragmentagáo das agóes e condutas profissionais em resposta aos comportamentos dos acompanhantes das criangas durante a internagáo hospitalar numa perspectiva de redugáo da dicotomia assistencial e reuniáo de condutas terapeuticas compartilhadas que favoregam a seguranga e o enfrentamento da PVP.

A realizagáo desse estudo se justifica na necessidade: 1) de uma releitura na perspectiva dos sujeitos sociais que compartilham vivencias e experiencias de um procedimento (des)conhecido e doloroso; 2) de reconhecer o significado e a forma de enfrentamento dos acompanhantes e profissionais da equipe de enfermagem diante da PVP pediátrica para convergir esforgos e redimensionar um cuidado efetivo e menos traumático, congruente com o contexto e necessidades infantis^2^ e que almeje a seguranga, o enfrentamento eficaz e as condigóes para o crescimento e desenvolvimento da crianga; e 3) de aproximagáo das percepgóes grupais sobre o procedimento rotineiro da PVP nas terapeuticas pediátricas em um cenário socialmente constituído^6^^,^^8^. Justificando-se assim a espessura social do fenomeno para os componentes do grupo social (acompanhantes e profissionais de enfermagem).

Esta pesquisa teve como pressuposto o fato de os profissionais de enfermagem atuantes em setores pediátricos e os acompanhantes destas criangas, ao cuidarem, vivenciarem e compartilharem do mesmo contexto social (ambiente terapéutico hospitalar) durante o processo de PVP^2^, formarem um grupo socialmente constituído, visto sua “espessura social” a ponto de, serem capazes de ancorarem e objetivarem o objeto investigado^7^^,^^8^ que é PVP realizada em crianzas hospitalizadas enquanto "pertenga social” do mesmo grupo de cuidadores da Crianza em relagao ao procedimento de PVP.

Objetivou-se analisar as representagóes sociais dos profissionais de enfermagem e dos acompanhantes sobre a PVP realizada em criangas hospitalizadas.

## Materiais e Métodos

Investigagao qualitativa com triangulagao de dados delineada no referencial teórico- metodológico da TRS conciliando as abordagens estrutural^7^ e processual^9^. Foi atendido o protocolo *Consolidated Criteria For Reporting Qualitative Research* (COREQ).

Foi utilizado um instrumento de coleta de dados estruturado em: a) caracterizagao dos participantes; b) evocagóes livres nao hierarquizadas (abordagem estrutural da TRS) e c) entrevistas individuais em profundidade gravadas (abordagem processual da TRS).

A investigagao foi desenvolvida no setor de pediatria de um Hospital Geral de Minas Gerais, Brasil, com a participagao de 118 acompanhantes das criangas hospitalizadas e submetidas a PVP e 22 profissionais de enfermagem em ambas as abordagens da TRS. Cabe mencionar que, nessa investigagao o acompanhante da crianga foi concebido como a pessoa que estava presente em tempo integral junto a ela durante o período de hospitalizagao, considerada a cuidadora principal e significativa por vínculo consanguíneo/afetivo.

Para os acompanhantes das criangas hospitalizadas o delineamento amostral baseou-se nas recomendagóes para estudos em TRS na abordagem estrutural (n >100^10)^ e para os profissionais de enfermagem foram incluídos todos os elegíveis (N=22). Tal fato justifica pela análise prototípica ser estratégia que organiza dados de acordo com a finalidade da pesquisa, nao configurando em uma técnica de análise estatística inferencial que necessita de uma amostra com tamanho mínimo para sua realizagao efetiva pois há evidéncias de que outras técnicas de abordagem estruturais na TRS também possuam pontos limitantes e facilitadores,^11^^-^^13^ sendo a triangulagao de técnicas e métodos (abordagens processual e estrutural) uma estratégia utilizada para minimizá-las^14^^-^^17^.

O recrutamento foi realizado pela pesquisadora principal no setor de pediatria, após ambiéncia e insergao no cenário de investigagao, no qual realizou o convite individual aos potenciais participantes e a explicitagao dos objetivos, potenciais benefícios e riscos e esclarecimentos de dúvidas. A aquiescéncia foi registrada por assinatura do Termo de Consentimento Livre e Esclarecida (TCLE) pós-informado. Houve duas perdas de profissionais que estavam de licenga médica, nao havendo recusa ou desisténcia em participar.

Foram critérios de elegibilidade: a) ser acompanhantes das criangas hospitalizadas e em uso de PVP e b) ser profissionais de enfermagem (enfermeiros, técnicos/auxiliares) atuantes no setor pediátrico da instituigao de todos os turnos/plantóes. Foram excluídos: acompanhantes que nao possuíam cognigao compatível com a abordagem em profundidade e/ou nao tinham presenciado a PVP na crianga e os profissionais que estavam de férias ou licenga médica.

*A* abordagem estrutural da TRS precedeu as demais etapas de coleta e organizado dos dados, a fim dessas nao influenciarem nos dados produzidos. Ela foi realizada a partir da aplicado da técnica da associagáo livre de palavras nao hierarquizada, sendo solicitado aos participantes que mencionassem as cinco primeiras palavras que lhes viessem a mente ao ouvirem o termo indutor “pegar veia de uma crianza", cujo registro foi documentado na ordem de mengáo.

Foram questóes norteadoras na abordagem processual da TRS: Conte-me um caso sobre pegar veia (puncionar veia) que tenha ocorrido com sua(uma) crianga, que o(a) Sr(a) se lembre. Como é para o(a) Sr(a) quando pegam (puncionam) a veia da sua(uma) crianga? O que vocé conhece sobre pegar veia em criangas? Se o(a) Sr(a) fosse representar a pungáo de veia numa crianga qual objeto o(a) Sr(a) usaria? Como sua(as) crianga(s) reage(m) quando pega(m) a veia dela(s)?

Os dados da abordagem estrutural e de caracterizagáo sociodemográfica foram coletados e registrados com auxílio do aplicativo *Open Data Kit* (ODK) reduzindo-se assim possíveis *vieses* de digitagáo. O processo de coleta de dados dos acompanhantes foi realizado pela pesquisadora principal, no leito da crianga e dos profissionais de enfermagem individualmente em sala reservada, no período compreendido entre abril/setembro de 2018 com duragáo de ±60 minutos.

Os dados de caracterizagáo foram consolidados e analisados com apoio do software *Statistical Package for the Social Sciences* (SPSS) versáo 26 e tratados com estatísticas descritivas (medida de dispersáo e centralidade). Os cognemas evocados foram consolidados para a elaboragáo do dicionário de termos equivalentes, utilizando a técnica lexicográfica para posterior análise prototípica. O *corpus* foi tratado no software *Ensemble de Programmes PemettantL'Analysedes Evoctions (EVOC) 2005* que forneceu o quadro de quatro casas. Foram contabilizadas: 526 e 105 palavras evocadas, com 56 e 39 cognemas diferentes, respectivamente para os acompanhantes e profissionais. Foi padronizado a média das ordens médias (*Rang*) de evocagáo em 2,6. Como o número de participantes dos segmentos sociais eram distintos foram adotados os seguintes parámetros na análise prototípica: 12 e trés; e 20 e seis como frequéncia mínima e média, respectivamente para acompanhantes e profissionais. A base de dados foi armazenada no Mendeley Data^18^.

A ordenagáo dos cognemas evocados ocorreu a partir dos critérios de frequéncia, *Rang* e Ordem Média de Evocagáo (OME) cuja alocagáo foi estruturada em quatro quadrantes possibilitando a interpretagáo do processo hierárquico e dos conteúdos das RS. No quadrante superior esquerdo (QSE), o provável núcleo central, foram alocados os cognemas evocados com maior frequéncia, menor *Rang* e menor OME, retratando conteúdos consensualizados pelo grupo social. No quadrante superior direito (QSD), a primeira periferia, estáo as evocagóes de maior frequéncia, *Rang* e OME altos que configuram comportamentos, informagóes, valores ou objetos traduzindo posicionamentos individuais e náo de grupo. No quadrante inferior direito (QID), a segunda periferia, estáo localizados os cognemas evocados de baixa frequéncia, *Rang* e OME altos, que possuem fungáo de estabilizar o conteúdo do núcleo central. E no quadrante inferior esquerdo (QIE), a área de contraste, foram alocados os cognemas de menores frequéncias, *Rang* e OME, contrastando com o conteúdo do núcleo central pela frequéncia, mas com potencial de ascensáo ao núcleo da representagáo devido os valores de *Rang.*

Os conteúdos discursivos (abordagem processual) advindos das entrevistas em profundidade constituíram o *corpus,* sendo tal conteúdo transcrito na íntegra no programa *Word for Windows 2016.* Para identificagáo das regras da exaustividade, representatividade, pertinéncia e homogeneidade procedeu-se a leitura de todo o *corpus* em profundidade, inserindo-o no software *Nvivo Pro11®*. Foram percorridas as seguintes etapas metodológicas para a análise de conteúdo: pré-análise, exploragáo do material e tratamento dos resultados, inferencia e interpretado^18^ e utilizadas categorías predefinidas (alocadas em “nós”- unidades de análise) segundo a análise de conteúdo temático-categorial, tendo por base as configurares das unidades de análise e registro na configurado das categorias^19^^-^^20^.

Os critérios adotados para definido das categorias predefinidas foram: as dimensóes (comportamentais/atitudinais, informativa/cognitiva, valorativa/afetiva e objetival/imagética) e as origens dos conteúdos representacionais (experiencias pessoais; com terceiros, familiares, acompanhantes, etc.). Para reunir as unidades de análise e registro na configurado das categorias foram agregados ou separados conteúdos dos *corpus* e alocados nos “nós”, utilizando como critério valores de Pearson > 0,7 na busca pelo equilibrio interno e homogeneidade entre as estruturas categoriais.

Desse procedimento técnico foi possível identificar que emergiram duas categorias. A análise realizada no *NVivo Pro11®* possibilitou identificar 118 e 22 fontes e 1102 e 456 Unidades de Registro (URs), respectivamente para os acompanhantes e profissionais de enfermagem.

Cabe mencionar ainda que, da conciliado das abordagens representacionais com os cognemas evocados de forma triangulada com as duas categorias favoreceu a identificado das fungóes essenciais da TRS dentre os conteúdos representacionais: 1) saber- permite compreender e explicar a realidade através dos conhecimentos adquiridos; 2) identitária- define as características específicas do grupo, permitindo a elaboragáo de uma identidade social; 3) orientagáo- orientam os comportamentos e as práticas do grupo social; e 4) justificatória- justificam o posicionamento e os comportamentos dos atores sociais em um dado contexto^9^.

Foram atendidos todos os aspectos ético-legais em pesquisa envolvendo seres humanos, sendo a investigagáo aprovada em Comite de Ética em Pesquisa sobre o parecer consubstanciado n° 2.543.592, de 14/03/2018). Foram garantidos o anonimato/sigilo dos participantes com o uso de códigos alfanuméricos (ex: AC012 e PR020) para acompanhantes e profissionais respectivamente.

## Resultados

No perfil dos profissionais predominou: 20 mulheres (90,9%); 11 solteiras (50%); 15 com filhos (68,2%); 13 delas com idade de 31 a 50 anos (59,1%, variabilidade de 23-57 anos e média 42 anos); brancas (8) e pardas (8) (72,8%); 14 católicas (63,6%); 15 técnicas em enfermagem (68,2%), quatro enfermeiras e tres auxiliares de enfermagem; dez com tempos de atuagáo dez a 20 anos na enfermagem (45% e média 17,1%); e 15 delas com um a 20 anos na pediatria (63,4% e média 12,7%).

**Figura 1:**
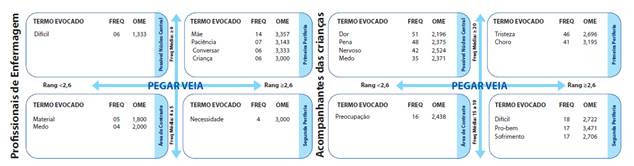
Esquema representativo dos quadros de quatro casas dos participantes: profissionais de enfermagem (A- n=22) e acompanhantes (B- n=118). Juiz de Fora (MG), Brasil, 2021.

No perfil dos acompanhantes das criangas caracterizou-se por: 103 mulheres (87,3%); 53 solteiras (44,9%); 115 com filhos (97,5%); 74 delas com idade entre 21 a 40 anos (69,7%, variabilidade de 18-65 anos e média 32 anos); 45 pardas (38,1%); 54 católicas (45,8%); 68 com escolaridade de nove a 12 anos (57,7% e variabilidade nove a 16 anos); 106 com renda entre uma a tres salários mínimos (90%); 86 com vínculo maternal com a crianga (72,8%); 116 delas com relato de experiencia com a PVP (98,3%, quer seja para infusáo ou coleta de material) e 91 com período de internagáo da crianga entre uma a sete dias (77,2%). Na figura 1 foram apresentados os quadros de quatro casas sobre “pegar veia” (abordagem estrutural da TRS) segundo os profissionais de enfermagem e acompanhantes das crianzas. Na análise prototípica, foi possível obter o quadro de quatro casas (Figura 1 A e B).

*Fonte: Dados da pesquisa. Nota: conteúdos extraídos do Programa Evoc 2005*.

Foram identificadas duas categorias na abordagem processual: 1) Dificuldades enfrentadas pelos profissionais na PVP em changas ao compartilhar o cuidado com a mae e 2) Sentimentos e comportamentos manifestados pelos acompanhantes frente a PVP nas criangas, conforme evidenciadas nas URs dos participantes (Quadro 1).

A análise de conteúdo temático categorial consta no Quadro 1.

A categoria “Dificuldades enfrentadas pelos profissionais na PVP em criangas ao compartilhar o cuidado com a mae” retratou conteúdos representacionais da abordagem processual que cor- roboraram com a estrutura representacional expressa pelo cognema valorativo *difícil* presente no possível núcleo central (Figura 1), conforme consta nas URs PR005 e PR011 (Quadro 1). Na área de contraste emergiu o sentimento de *medo* justificando a dificuldade na realizagao da PVP e o objeto *material* retratou a forma de instrumentalizagao do procedimento (PR004 e PR011- Quadro 1).

Na primeira periferia os cognemas *máe* e *crianza* foram objetos representacionais interligados e vinculados a representagao de ser *difícil,* podendo ainda considerar o cognema *máe* como um elemento periférico superativado visto sua alta frequéncia de evocagao, fato corroborado pelas URs (PR001, PR002 e PR014- Quadro 1). A presenga dos comportamentos periféricos *paciencia* e *conversar*, essenciais ao éxito do procedimento e a *necessidade*, como um valor justificatório da PVP corroborados pela abordagem processual (PR003 e PR009 - Quadro 1).

A categoria “Sentimentos e comportamentos manifestados pelos acompanhantes frente a PVP nas criangas” aproximada da análise prototípica (Figura 1) apontou que as RS foram objetiva das pela *dor* (possível núcleo central) ancorada nos sentimentos de *pena*, *nervoso*, *medo*, *preo- cupagáo* (área de contraste) e *tristeza* (primeira periferia), cuja percepgao foi mensurada pelo comportamento de *choro* das criangas (primeira periferia). Esses cognemas reforgam o possível núcleo central dando-lhe sentido e agregando valor social (ancoragem) que foram reafirmados pela abordagem processual (AC003, AC008, AC010, AC015 e AC023 **-**Quadro 1).

Os elementos periféricos: *difícil, pro-bem* e *sofrimento* expressam valores e sentimentos dos acompanhantes (Figura 1) oriundos do compartilhamento de experiencias entre o binomio profissional-acompanhante, corroborado pela abordagem processual AC004 e AC043 (Quadro 1).

**Quadro 1:** Esquema representativo da análise de conteúdo dos participantes: profissio- nais de enfermagem (A- n=22) e acompanhantes (B- n=118). Juiz de Fora (MG), Brasil, 2021.

	Categoria 1: Di_culdades enfrentadas pelos pro_ssionais na PVP em crianças ao compartilhar o cuidado com a mãe	Categoria 2: Sentimentos e comportamentos manifestados pelos acompanhantes frente a PVP nas crianças
Dimensões representacionais	COMPORTAMENTAL-ATITUDINAL: *Além de você puncionar, o que a gente comenta muito aqui é que você lida com a ansiedade da criança, com choro, com medo e o pior, as mães. Elas acham que você está fazendo a criança chorar... Judiando. PR004 Ah! As crianças, os grandinhos são mais... A gente tem um pouco mais de trabalho, né? Tem que ter um diálogo bom, muita paciência... Muita paciência! Não pode pegar logo, pegar o braço, pegar e furar e pronto não, sabe? Tem que conversar... PR009* COGNITIVA-INFORMATIVA: *Se o médico pedir pra tentar, a gente tenta quantas vezes o médico pedir. E quando é assim, a gente pede para o médico ficar presente, conversa com a mãe, explica que é para o bem da criança, que é necessário, deixa a criança descansar um pouco e tenta de novo. PR003* VALORATIVA: *Uma criança difícil de veia é muito ruim. Você vê que ela está precisando do acesso e você não consegue. Isso tem acontecido bastante aqui! É um procedimento demorado, porque a gente precisa de uma pessoa pra segurar, e muitas vezes, a mãe tem que estar junto, porque a criança não fica quieta. Se for uma criança maior, tem que segurar as perninhas, outra o bracinho e alguém pra poder fazer a punção. No momento que está fazendo a punção, a criança mexe o braço e depois que puncionou uma das pessoas que está ajudando a segurar, acaba fixando (cateter). Uma pessoa sozinha não consegue. PR011 Tem até uma criança que o acesso perdeu hoje e tentamos várias vezes e não consegui-mos. Está sem acesso! Ligamos pra médica assistente e ela falou que tem que puncionar. A gente faz revezamento (equipe). Olha!... A noite que passou foram 12 tentativas, Até agora, com a gente foram 6. Não tem condição mesmo! Não tem! E tem que pegar porque precisa da veia antes de ir embora. PR005* OBJETIVAL: *Só mesmo, o escalpe ou o cateter venoso. Eu falo escalpe porque tem lugares que não são padronizados, aqui mesmo não é... Mas o escalpe é usado porque é mais rápido. PR011 Porque puncionar a veia de uma criança já é um pouquinho mais complicado e se a mãe não estiver ajudando... Aí a coisa é pior. PR001 Bom... Criança... Vou ser honesta com você. Criança não é o problema para a gente. O problema nosso é a mãe! Tem mãe que maltrata muito a gente. PR014 Tem que prestar atenção na criança e, também na mãe. Saber como ela tá se sentindo. Teve uma vez que a gente estava puncionando e a mãe desmaiou e aí a gente não sabia se acudia a criança na maca ou a mãe. PR002*	COMPORTAMENTAL-ATITUDINAL: *Da pena! Dá, vontade de pedir pra não fazer. AC015 Eu reagi muito mal porque a moça não estava conseguindo pegar a veia, estava insistindo... Teve que transferir ela para a outra moça tentar. Eu fiquei muito nervosa porque eu estava falando que ela não estava conseguindo e ela tentando e a menina chorando e eu mais nervosa ainda. Tentaram umas duas vezes e ficou mexendo com a agulha dentro da menina. Nossa... É muito ruim isso. A criança sofre... AC023 Vem muito sangue! Tenho medo de não conseguir pegar veia e ficar sangrando... AC008 Eu fiquei um pouco apavorada em relação a ela, por ela ser novinha e me arrasou. Chorei junto com ela, fiquei chateada e triste. AC003 Preocupada! Porque nunca tinha visto e ela chorando me desesperou... AC010 Não tem outro jeito, né? Se tivesse outra forma que não tivesse que furar seria melhor. A criança não está acostumada com isso! Não sabe controlar a dor e entender isso. Mas não tem outra forma e é a melhor solução pra sarar mais rápido... Melhor sofrer rapidinho agora do que ficar sofrendo pra sempre, né? A gente sabe que é pro bem dele AC004* COGNITIVA-INFORMATIVA: *Ele chora e faz muita força e pra segurar ele precisa de umas 3 ou 4 pessoas, porque ele pede socorro e pede ajuda e pra não deixar fazer e fica desesperado. AC002 Preocupada! Porque eu nunca tinha visto e ela chorando me desesperou... AC010 Não conheço muita coisa não. A gente só conhece de ver eles fazendo mesmo. Mas de informação a gente não tem não AC002* VALORATIVA: *Não conseguiram pegar a veia dele, muito difícil e tentaram, nossa... Umas cinco ou dez vezes... Tentaram de noite e de dia umas 4 ou 5 profissionais. Tentaram de noite e deu. Aí de dia a veia dele arrebentou e perdeu e aí eu falei desisto, tadinho. Ele ficou todo inchadinho por causa disso. Ele sofreu pra caramba! Aí eu falei: Ah! Não. Eu não quero que meu filho sofra não! Chega! Chega! Tá doido! AC043 É ruim quando não consegue e tem que ficar espetando a criança e você fica olhando assim e dá dó e vontade de sair correndo. AC005 É horrível! Ah... É muito triste! Você não pode fazer nada e sua filha está sentindo dor. AC006.* OBJETIVAL: *Para te falar a verdade, tive vontade na hora de pegar ela (criança) e ir embora. A hora que vi ela chorando, desesperada e tremendo e teve uma hora que a enfermeira precisou pegar ela e abrir a mão dela e ela fechou a mão gritando e chorando, porque ela é brava...Eu tive vontade de pegar ela e as coisas dela e ir embora, mas a gente tem que pensar no bem estar, né? AC003*
Origens representacionais	PRÓPRIA: *Tem que prestar atenção na criança e, também na mãe como ela tá se sentindo. Teve uma vez que a gente estava puncio-nando e a mãe desmaiou e aí a gente não sabia se acudia a criança em cima da maca ou se acudia a mãe.PR002* 128. CONHECIDOS: *Já teve uma veia que foi pega e que era artéria. A criança ficou toda roxa, o rostinho e o bracinho. Cheia de petéquias. Foi no braço e na mesma hora que puncionou e tirou na mesma hora. PR007* 129. MÃE-ACOMPANHANTE: *A gente orienta a mãe e desde que ela entre na sala e não fique atrapalhando, querendo pegar a criança na “cachola”, falando que já chega, que está furando muito. PR004 A mãe já está chorando, então a criança já está vendo a mãe chorar por causa de puncionar a veia e atrapalha. [...] Dificuldade que existe mesmo é o nervosismo até dos pais que atrapalha. PR006* 130. PROFISSIONAIS: *A gente chama até a enfermeira para conversar, a gente tenta conversar primeiro, mas quando elas (mães) não escutam, a gente chama a enfermeira pra conversar. Aí, quando não deixa mesmo, pede o médico para mandar medicação via oral. PR013*	PRÓPRIA DO FAMILIAR/ACOMPANHANTE: *Eu fico nervosa porque eles (crianças) ficam nervosos e me colocam nervosa. O que eles sentem eu sinto. Não brigo nem nada não porque muita mãe não tem paciência. Eu procuro ser o mais tranquilo possível. AC008 Eu fiquei um pouco apavorada em relação a ela, mesmo por ela ser novinha e me arrasou. Chorei junto com ela e fiquei chateada e triste. AC003* CRIANÇA: *Mas eu me sinto mal. Se você vê que ele tá chorando é porque tá doendo. Aí me sinto mal de ver ele sofrendo, né? AC016 Foi só uma que eu chorei porque tentou e foi muitas vezes e ele não estava nem chorando mais, de não estar aguentando. AC001* CONHECIDOS: *A gente estava comentando de um caso de fora. De uma moça que pegou a veia de um bebê no hospital. Furou a veinha e em vez de olhar vez curativo e deu um monte de proble-mas no pé do bebê AC011* PROFISSIONAL: *teve uma hora que a enfermeira precisou pegar ela e abrir a mão dela e ela fechou a mão gritando e chorando, porque ela é brava...Eu tive vontade de pegar ela e as coisas dela e ir embora, mas a gente tem que pensar no bem estar, né? AC003 O que eu entendo é que eles falam, geralmente, é que faz mais efeito pela veia que é bem mais rápido AC024*

*Fonte: Dados da pesquisa. Nota: conteúdos extraídos do Programa Nvivo Pró11®.*

## Discussao

Na análise concomitante dos constructos representacionais (abordagens estrutural e proces- sual) o termo *difícil,* de dimensáo valorativa emergiu no possível núcleo central para os profis- sionais de enfermagem e na segunda periferia para os acompanhantes, retratando a dimensao valorativa ao determinar a avaliagáo ou julgamento sobre o procedimento executado e fungáo justificatória da TRS ao representar o conceito socialmente compartilhado pelos profissionais de enfermagem de que se trata de um procedimento de difícil execugáo, corroborado pelas URs dos profissionais de enfermagem (PR011 e PR005) e dos acompanhantes (AC005) (Quadro 1).

O ato de pegar a veia de crianzas pelos profissionais de enfermagem foi avaliado como difícil, porém, necessário e influenciado por diversos fatores como a presenta, aceitagáo, sofrimento (choro e dor) e nervoso dos acompanhantes e da crianza; as diversas tentativas para éxito; a idade e a agitagáo da crianza. A dificuldade em se estabelecer um acesso Intravenoso (IV) por um profissional qualificado e experiente está relacionado a visibilidade e a palpabilidade da rede venosa, idade, prematuridade e tonalidade da pele além de questóes físicas, psicológicas, pessoais e interpessoais, tipo de material utilizado e terapéutica instituída^21^.

Os profissionais de enfermagem se sentem inseguros em realizar o procedimento de PVP, dian te das dificuldades, caracterizada pelas especificidades da rede venosa de difícil visualizado e palpado; a pouca idade, sendo maior a dificuldade quanto menor for a crianza; acrescido do período de desenvolvimento cognitivo que ela se encontra o que gera agitado, náo aceitado e agressóes físicas/verbais aos profissionais que executam o procedimento somado a presenta dos acompanhantes que pode contribuir ou dificultar na imobilizagáo e convencimento da crianza^4^^,^^21^.

Na zona de contraste para os profissionais de enfermagem, o cognema *material* (Figura 1), com dimensáo representacional objetival e fundo identitária e justificatória, demonstrou o caráter de cientificidade e especificidade do procedimento, remetendo a técnica de realizado e a ne- cessidade de tecnologias especializadas quando os profissionais sáo levados a assegurar o éxito do procedimento, caracterizando a preocupado inicial, o qual se tem que ter atengáo. Essa constatado é exemplificada nas falas dos participantes (PR001 e PR0011 - Quadro 1).

Os materiais utilizados para viabilizar a PVP ou facilitar a sua execugáo sáo identificados pelo cateter, escalpe, leito/maca, bergo, mesa e sala de pungáo. Para a realizagáo da PVP é necessário, primeiramente, o preparo do material evitando idas e vindas durante o procedimento por es- quecimento de algo o que pode gerar inseguranga na pessoa a ser puncionada e seu familiar, uma imagem de desorganizagáo do servigo, desgaste físico do profissional e aumentar o tempo de realizagáo do procedimento o que aumenta também o estresse ao procedimento^4^^,^^22^.

Uma revisáo integrativa realizada com objetivo de caracterizar os passos da técnica de PVP por profissionais de saúde apontou que dentre os 12 artigos inclusos sobre a temática, sete citavam o *material* como item presente nas observagóes iniciais dos profissionais na etapa que antecede o procedimento^23^. Este dado é confirmado com o cognema mencionado pelos profissionais de enfermagem que ficou alocado na área de contraste do quadro de quatro casas. Ele retrata que esse conteúdo foi mencionado prontamente nas primeiras posigóes (OME=1,8), na medida em que remete aos equipamentos necessários utilizados pelos profissionais ao realizarem a PVP e, que é capaz de influenciar no éxito do procedimento. A potencialidade desse conteúdo ascen-der ao núcleo central da representado social se deve ao seu valor de *Rang, e* depende que sua frequéncia seja aumentada na medida em que for consensualizada entre no grupo social^8^.

Na abordagem processual foi mencionada a preocupado do profissional com o material, sendo uma das primeiras etapas do procedimento de PVP, por poder minimizar a dificuldade e reque rer o atendimento da especificidade dessa técnica quando realizado na crianza; fato corrobora pelos conteúdos mencionados pelos participantes PR005, PR011 e PR004.

Dentre os acompanhantes das crianzas, no possível núcleo central emergiram os cognemas, *dor, pena, nervoso* e *medo* todos componentes da dimensao comportamental e fundo de orien tado e justificatória. Essa RS é mencionada pelos acompanhantes das crianzas AC003, AC004, AC008, AC010, AC015 e AC023 (Quadro 1). Os acompanhantes justificam seus comportamentos como consequéncia do auxilio na colaborado da crianza visando o éxito da PVP, dessa forma, elas tentam controlar seus sentimentos e as reagóes negativas das crianzas manifestadas por dor e medo frente a necessidade da realizado da PVP. Porém os comportamentos das criangas refletem no de seus acompanhantes que reagem com sentimentos de pena e nervoso quando avaliam a competéncia do profissional mediante as suas atitudes, habilidades, dificuldades e éxitos durante a execugao do procedimento.

As RSs da PVP pelos acompanhantes das criangas se aproximam da perspectiva de outros grupos sociais como adultos hospitalizados, profissionais de enfermagem atuantes em setor pediátrico ao remeteram respostas humanas negativas oriundas da experiéncia pessoal da observagao de vivéncias de pessoas hospitalizadas e do contato com os profissionais. Houveram relatos que caracterizaram sentimentos e comportamentos negativos expressos por medo, dor, sofrimento, desconforto e nervosismo, acrescidos de incomodo, inseguranga, ansiedade, choro, dúvida, pavor, restrigao de movimentos e submissao ao profissional executor da PVP, sendo neste grupo social aproximado também a fungao justificatória do procedimento como indispensáveis ao tratamento e reabilitagao clínica^24^^-^^27^

A dor, uma reagao ao medo do ato de perfurar a veia com uma agulha, é uma ocorréncia corri- queira e sua prevengao deve ser encorajada, visto a complexidade desta sensagao e por englo bar elementos multidimensionais referentes a própria crianga, profissionais e aos pais^28^^,^^29^.

A pena foi compreendida como um sentimento de misericórdia, representando o cuidado, preocupagao com o outro no momento da PVP diante do medo e do choro das criangas e da atuagao dos profissionais de enfermagem que assistem/cuidam em diferentes condigóes de tratamento/reabilitagao e níveis de sofrimento^3^^,^^26^. O nervoso, sentimento do acompanhante frente ao sofrimento repetitivo da crianga ocorre pela perda do papel de cuidador durante a hospitalizagao, o que culmina com comportamentos agressivos e negagao dos procedimentos ameagadores^33^.

O termo *medo* presente no possível núcleo central dos acompanhantes e na área de contraste dos profissionais de enfermagem, foi um comportamento expresso com importancia diferenciada, capaz de fortalecer o possível núcleo central dos profissionais de enfermagem ressaltando as dificuldades ao puncionar criangas (Figura 1). O medo se refere ao sentimento da crianga relacionado ao procedimento, que gera comportamentos/atitudes como agitagao, choro e negagao ao procedimento. Este é evidenciado como o sentimento predominante nos profissionais de enfermagem ao puncionar criangas e foi corroborado por outras investigagóes^2^^,^^29^.

*O* medo é definido como uma resposta produzida por uma informado que vem do mundo externo (o procedimento de PVP) ou interno as crianzas (sentimento) que é valorado negativamente, automático, inconsciente e preparam a crianza para agir diante do perigo e estresse da necessidade da PVP. As crianzas de acordo com a idade ainda nao sabem lidar e controlar suas emogóes e assim reagem de formas inadequadas, agressivas, verbal e/ou fisicamente contra o profissional que o punciona ou o imobiliza para a realizagao do procedimento como forma de negar a ocorréncia dessa situagao desconhecida e desconfortável para ela^2^.

Na zona de contraste dos acompanhantes das criangas identificou-se o termo *preocupando,* que representou a dimensao comportamental e fungao do saber conforme apresentado no relato do acompanhante (AC010 - Quadro 1). A preocupagao é um sentimento dos acompanhantes frente a (des) informagao a respeito dos procedimentos/tratamentos realizados na crianga durante a terapéutica implementada e internagao hospitalar. Desse modo, reforga-se a necessidade de inclusao do familiar/acompanhante no cuidado a crianga, compartilhando informagóes necessárias para o enfrentamento eficaz ao procedimento e planejamento de agóes de cuidado^2^^), (^^30^.

Existe na abordagem estrutural da TRS a possibilidade dos elementos alocados na primeira periferia serem centrais, por seu caráter quantitativo, quando empregados critérios como conexidade ou afinidade com outros elementos centrais.^7^ Nesse sentido, o termo *mde* foi identificado como o cognema com maior frequéncia (14 evocagóes) (Figura 1), configurando sua importancia representacional sobre PVP pelos profissionais e sendo considerado, portanto um elemento periférico superativado, na dimensao objetival e com capacidade de refletir as quatro fungóes representacionais a medida que a presenga da mae durante a PVP pode ser exitosa ou dificultadora quando assume ou nao a modulagao do enfrentamento da crianga, conforme descrito pelo profissionais PR001, PR002 e PR0014 (Quadro 1).

Dessa forma, a presenga do termo *mde* em todo o processo de PVP, como possível núcleo central, complementaria o significado representacional de “pegar veia”. Assim, a RS de pegar veia pelos profissionais de enfermagem foi objetivada ao retratarem uma “imagem” ao objeto representacional e ancorada ao classificar o procedimento como difícil.

Os profissionais identificam a mae, presente durante o procedimento, de forma dicotomica sendo (des) favorável. Houve relatos de ajuda e em contraponto reagóes negativas durante o procedimento (negagao, resisténcia e desmaios) que reforga a dificuldade de realizagao do procedimento (Quadro 1). Evidencia-se uma dificuldade em inserir a família no cuidado das criangas, apesar do familiar ser fundamental como apoio a crianga (processo de significagao)^30^.

As criangas, identificadas na primeira periferia dos profissionais de enfermagem, sao caracterizadas como outro dificultador a realizagao da PVP, sendo um elemento representacional capaz de fortalecer/protege o núcleo central. As criangas vivenciam períodos de desenvolvimento psicossocial e cognitivo que geram comportamentos instáveis e imprevisíveis frente a necessidade de PVP, pois cada crianga apresenta velocidade de desenvolvimento diferenciado, individual a cada ser que pode ser influenciado pela sua cultura, ambiente, estímulos e genética^28^.

Os demais cognemas da primeira periferia dos profissionais de enfermagem reforgam o possível núcleo central por caracterizarem a PVP como um procedimento *difícil* que precisa ter *paciencia* ao realizá-lo e requer diálogo e orientagao com os acompanhantes da crianga para o éxito do procedimento. O cognema *paciencia* permeia a conduta laboral quando lida com o medo das crianzas e com a ameaga percebida no comportamento dos acompanhantes durante o procedimento. Isso requer habilidade ao lidar com ambos o que justifica a percepgao dicotomica do profissional quanto a contribuido da presenga dos pais durante a PVP^2^^-^^3^.

A presenga dos cognemas *conversar* (fungao orientadora), *paciencia* (fungao justificadora) e *crianza* (fungao identitária) sao corroborados na abordagem processual na dimensao comportamental e objetival que expressam a necessidade de os profissionais esclarecerem a razao do procedimento, acalmar a crianga e inserir o acompanhante no cuidado, uma vez que, a experiencia junto a eles foi considerada um ponto dificultador **(**PR009 e PR014 - Quadro 1).

As fungóes orientadora e justificadora desses cognemas remetem a necessidade de corrobo- ragao e aceitagao da díade crianga-acompanhante melhorando o enfrentamento dos mesmos frente a PVP e da hospitalizagao, o que retrata o redimensionamento do planejamento da as- sistencia de enfermagem^25^; caracterizada por ser desafiadora por ser estabelecida diariamente em busca de vínculos e confianga pautados no diálogo e na paciencia para uma assistencia humanizada^29^.

Na segunda periferia estao os cognemas menos frequentes (F<6) e que foram mais tardiamente evocados (OME>2,1). Nela emergiu o termo *necessidad*e para os profissionais e, *difícil*, *pro-bem*, (dimensao valorativa e fungao justificadora) e *sofrimento* (dimensao comportamental e fungao identitária) para os acompanhantes das criangas (Figura 1). Estes foram considerados os menos importantes para a determinagao do significado da RS abarcando aspectos relativos a realida de individual dos sujeitos baseado em suas vivencias,^7^^-^^9^ exemplificados e corroborados pelos fragmentos da abordagem processual segundo os profissionais de enfermagem (PR003, PR004, PR005 e PR011 - Quadro 1).

Os cognemas periféricos mencionados pelos acompanhantes (Figura 1) expressam que, o *choro* da crianga ocorre devido a *dor* e ao *medo* do desconhecido, decorrentes da PVP. Os acompanhantes relataram *tristeza* como um sentimento derivado de sua proximidade e vínculo com a crianga frente as dificuldades na PVP. A atuagao da equipe de enfermagem frente a esses sentimentos objetiva melhorar a adaptagao da família frente as situagóes vividas durante a internagao e a PVP^31^. O ato de chorar é uma atitude de enfrentamento diante de situagóes estressoras para a crianga hospitalizada, ao gerar dor fisiológica que impacta sobre todos os envolvidos^28^.

Visto a necessidade e importancia de se viabilizar um Cateter Intravascular (CIV) pérvio em uma crianga hospitalizada para a farmacoterapia prescrita as criangas apresentam estranhamento diante do procedimento de PVP, vinculando-o a uma fonte de sofrimento, sendo a educagao para a saúde realizada pelo enfermeiro capaz de reduzir tensóes, estimular o cuidado e a aceitagao para o tratamento clínico-farmacológico humanizado que contribua para a tranquilidade dos envolvidos^30^.

Ao analisar as RS de pegar veia segundo os atores sociais investigados, entende-se que a identificagao da caracterizagao da dificuldade de pegar veia na crianga pelos acompanhantes nao é “consensualizada”, uma vez que, está presente o cognema “difícil” na segunda periferia com fungao justificadora que permite explicar como os acompanhantes atribuem as várias tentativas de PVP no público infantil ao despreparo e inexperiencia dos profissionais na medida em que nao conhecem as características cientificamente comprovadas dos fatores dificultadores de PVP em criangas.

*As* RS de “pegar veía" em crianzas pelos profissionais de enfermagem se estruturou sobre a dificuldade na realizado do procedimento acrescido da necessidade de insergáo da máe como coparticipante do cuidado com a crianza. O ato de pegar veia da crianza possui um impacto psicológico negativo para os acompanhantes, gerando medo, pena, dor e nervoso. Cabe ressaltar que o sentimento de medo das crianzas e dos acompanhantes foi identificado pelos profissionais de enfermagem como outro dificultador da instalado do CIV.

Portanto, devido o procedimento de PVP nas crianzas ser caracterizado como de difícil execugáo pelos profissionais de enfermagem que geralmente necessitam de mais de uma tentativa para se estabelecer o CIV, ele gera manifestares de medo da agulha, de dor física na crianza e psicológica no acompanhante, vinculando a sentimentos de nervoso e pena frente ao sofrimento da crianza e as reagóes de negagáo que apresentam frente as várias tentativas de PVP.

As RS dos acompanhantes das criangas foram permeadas na pena frente ao sofrimento experimentado/vivenciado pela crianga ao puncionarem sua veia. Seus comportamentos e sentimentos expressos por medo, nervoso e dor, aproximaram as RS da PVP identificado em outros grupos (pessoas adultas e criangas escolares) com o dos acompanhantes de criangas, porém ocorreu de modo diferenciado no público infantil pela presenga do sentimento de pena^24^^,^^33^. Estes dados proporcionam reflexóes sobre os sentimentos de sofrimento que estáo presentes nas RS das pessoas que possuem seus vasos puncionados, ou ainda, ao presenciarem outra pessoa puncionada com o qual possui vínculo afetivo^17^^,^^32^.

Estudo que identificou as RS da PVP em adultos hospitalizados descreveu a percepgáo desse procedimento, através de relatos de dor, medo, ansiedade e sofrimento^17^. Isso remete a com- preensáo de que os acompanhantes identificam as RS de PVP pelas criangas (dor e medo) seja por transferirem valores e experiencias próprias a ela e/ou por compartilharem a vivencia deste procedimento.

Os sentimentos dos acompanhantes acerca da PVP em criangas sáo resultados novos, náo sendo encontrados na literatura outros estudos que avaliassem as RS dos acompanhantes de criangas. Isso aponta a necessidade de incorporagáo de pesquisas que incluam o acompanhante como pessoa integram do cuidado compartilhado. O acompanhante está presente no ambiente hospitalar desde 1990 e até hoje negligenciado no que tange a sua insergáo na operacionalizagáo da PVP^30^.

Observou-se que a relagáo entre os profissionais que puncionam e o acompanhante da crianga puncionada é de percepgóes permutadas, isto é, as múltiplas PVP ocorrem no público infantil devido a dificuldade de realizar o procedimento, evidenciando representagóes que expressam sentimentos negativos por parte dos acompanhantes, enquanto grupo social^24^^,^^33^.

Os dados revelam que as RS da PVP pelos profissionais de enfermagem reduzem-se as técnicas de insergáo do CIV, negligenciando o cuidado centrado na família. Evidencias apontam para um *déficit* na formagáo profissional, acarretando desgaste emocional para as criangas, para a equipe e família. Sugere-se a utilizagáo de tecnologias que auxiliem na insergáo e manutengáo do CIV visando minimizar a fragmentagáo do cuidado a crianga e viabilizar a insergáo do acompanhante, ao instrumentalizá-los para o preparo em uma atuagáo conjunta no cuidado integral a crianga^4^^,^^17^^,^^25^^,^^30^.

Este fato também é apontado durante o cuidado de enfermagem na etapa de manutengo do acesso venoso em changas, em que prevalecem comportamentos tecnicistas com a preocupagáo voltada para a infusáo medicamentosa e a importancia da fixagáo para um acesso venoso pérvio contrapondo a representagáo social dos acompanhantes das criangas norteado por sofrimento, relatos de tristeza e a valoragáo da necessidade da procedimento^27^.

Foram consideradas possíveis limitagóes desta investigagáo a impossibilidade de transposigáo dos resultados para outras realidades pode ser justificada pelo reduzido número de profissionais de enfermagem (N). Para minimizar essas limitagóes fez-se a triangulagáo de abordagens (estrutural e processual).

Sáo contribuigóes para a área da enfermagem e saúde a identificagáo dos significados e sentimentos do processo de PVP em criangas numa perspectiva das RS de profissionais de enfermagem e dos acompanhantes das criangas hospitalizadas estimulam reflexóes sobre a abordagem profissional e a importancia de se inserir os acompanhantes, vínculos de estabilidade da crianga, após eles receberem apoio para o enfrentamento de um procedimento técnico, visto tratar-se de um conteúdo reificado para o qual ele necessita ser atendido em suas necessidades quando se pretende inseri-lo em um cuidado compartilhado, humanizado e específico ao grupo socialmente contextualizado.

## Conclusoes

Para os profissionais de enfermagem a PVP se estruturou sobre a dificuldade de realizagáo do procedimento e de se inserir a máe em um cuidado compartilhado, uma vez que sua forma de enfrentamento da PVP se mostrou segundo suas vivencias como um desafio adicional que se depara no exercício profissional. Para os acompanhantes as RS foram valoradas negativamente visto o impacto psicológico ao verem a crianga exposta a um procedimento doloroso, necessário e que requer um posicionamento participativo e colaborativo no cuidado compartilhado com a enfermagem.

Ao se refletir sobre a PVP pediátrica a luz das RS evidenciadas identificou-se como contribuigáo a necessidade de se repensar estratégias que favoregam o processo de enfrentamento da PVP pelo acompanhante quando se almeja seu engajamento no cuidado compartilhado.

## References

[1] Bitencourt ES, Leal CN, Boostel R, Mazza VDA, Felix JVC, Pedrolo E (2018). Prevalence of phlebitis related to the use of peripheral Intravenous devices in children. Cogitare Enferm.

[2] Ribeiro JP, Gomes GG, Thofehrn MB, Mota MS, Cardoso LS, Cecagno S hospitalized children: perspectives for the shared care between nursing and family (2017). Rev Enferm. UFSM.

[3] Buboltz FL, Silveira A, Neves ET, Silva JH, Carvalho JS, Zamberlan KC (2016). Family perception about their presence or not in a pediatric emergency situation. Texto Contexto Enferm.

[4] Infusion Nurses Society (2021). Infusion therapy standards of practice. J Infus Nurs.

[5] Ministério da Saúde (Br) (2018). Secretaria de Ciencia, Tecnología e Insumos Estratégicos. Departamento de Ciencia e Tecnologia. Agenda de prioridades de pesquisa do Ministério da Saúde - APPMS.

[6] Almeida TJC, Oliveira JFM, Santos LM, Santana RCB, Camargo CL, Sobrinho CLN (2016). Peripheral venous accesses in hospitalized children: a photographic study. Rev. Enferm. UFPE.

[7] Sá CP (2015). Estudos de psicologia social: historia, comportamento, representares e memória.

[8] Moscovici S (2017). Representares sociais: investigares em psicologia social.

[9] Abric JC (2013). Prácticas sociales y representaciones.

[10] Wolter R (2018). Structural Approach Theory and Method. Psico-USF.

[11] Wachelke J, Wolter R, Matos FR (2016). Effect of the size of the sample in the analysis of evocations for social representations. Liber.

[12] Sá CP (2002). Núcleo central das representares sociais.

[13] Abric JC (2007). Méthodes d'etude des representations sociales.

[14] Melo LD, Arreguy-Sena C, Gomes AMT, Parreira PMD, Pinto PF, Rocha JCCC (2020). Social representations elaborated by elderly people about being elderly or aged structural and procedural approaches. Rev. Enferm. UFSM.

[15] Gomes ICR, Lira MOSC, Rodrigues VP, Vilela ABA (2020). Representaciones sociales de mujeres en situaciones de violencia doméstica en la asistencia jurídica. Rev Cuidarte.

[16] Pereira APN, Arreguy-Sena C, Queiroz ABA, Dutra HS, Melo LD, Krempser P (2020). Social representations of primary care nurses on nursing records. Enfermagem Brasil,.

[17] Arreguy-Sena C, de Melo LD, Braga LM, Krempser P, Lemos RDCPB, Lopes DP (2019). Peripheral vein puncture in hospitalized adults nested sequential mixed method. Enfermagem Brasil.

[18] Krempser P, Caldas CP (2022). Banco_dados_RS_PVP_Família_Enfermagem, 2022. Mendeley Data, V1.

[19] Bardin L (2016). Análise de Conteúdo. Reimpressáo da Edigáo revista e actualizada de 2009.

[20] Oliveira DC (2016). metodologias de pesquisa para a enfermagem e saúde: da teoria para a prática.

[21] Freire MHS, Arreguy-Sena C, Muller PCS (2017). Cross-cultural adaptation and content and semantic validation of the Difficult Intravenous Access Score for pediatric use in Brazil Rev. Latino-Am. Enfermagem.

[22] Floriano CMF, Avelar AFM, Peterlini MAS (2019). Time-related factors for peripheral intravenous catheterization of critical children. Rev Bras Enferm.

[23] Lomba L, Gomes AC, Bogalho C, Jesus I, Sousa AF (2020). Prevention of complications in peripherally inserted central lines: an integrative review of the literature. Rev. Iberoam. Educ. Invest. Enferm.

[24] Krempser P, Caldas CP, Arreguy-Sena C, Melo LD (2020). Social representations and pediatric venus puncture stressors contributions to nursing care. Enferm. Foco.

[25] Campos LB, Martins JR, Arreguy-Sena C, Alves MS, Teixeira CV, Souza LC (2016). Experiences of hospitalized patients with the venipuncture process Esc. Anna Nery Rev. Enferm.

[26] Melo LD, Arreguy-Sena C, Krempse P, Braga LM, Gomes AMT (2021). Peripheral venipuncture in hospitalized people: GIBI technique supporting the procedural approach to social representations.

[27] Krempser P, Caldas CP, Arreguy-Sena C, Melo LD, Krepker FF (2020). Maintenance of peripheral venipuncture in children perspectives of nursing professionals and companions. Research, Society and Development,.

[28] Mendes-Neto JM, Santos SL (2020). Vibration associated with cryotherapy to relieve pain in children. BrJP.

[29] Gonalves KG, Figueiredo JR, Oliveira SX, Davim RMB, Camboim JCA, Camboim FEF (2017). Hospitalized child and the nursing team: opinion of caregivers. Rev. Enferm. UFPE.

[30] Silva CG, Santos L, Souza M, Passos S, Santos S (2020). Validación de cartilla sobre cateterización intravenosa periférica para familias Av. Enfermería.

[31] Krempser P, Arreguy-Sena C, Parreira PMSD, Salgueiro-Oliveira AS (2019). Nursing protocol in vascular trauma prevention peripheral catheterization bundle in urgency. Rev Bras Enferm.

[32] Antao C, Rodrigues N, Souza F, Anes E, Pereira A (2018). Hospitalization of the child parental feelings and opinions. Rev. INFAD de Psicología.

[33] Faciolli SC, Tacla MTGM, Candido LK, Ferrari RAP, Gabani FL (2017). Pungáo venosa periférica o olhar da crianza hospitalizada. REAS.

